# Supplementary material for: Accelerating covering array generation by combinatorial join for industry scale software testing
Source: PeerJ Comput Sci. 2022 Feb 11;8:e720. doi: 10.7717/peerj-cs.720 (PMC9044240; doi:10.7717/peerj-cs.720)
Supplement: Supplemental Information 1 — Describes the structure of the project, the contents of the Java project, and a way to reproduce our experiments and how the CASA data sets are organized. [file peerj-cs-08-720-s001.pdf]

README

This is a "readme" file for "supplemental materials" of our paper, "Accelerating covering array generation by combinatorial join for industry scale software testing".

The supplemental files are provided as `experiment.zip`. It contains a maven based Java project, raw experiment results, and their summaries. The project can be imported into your IDE such as IntelliJ or Eclipse and you can reproduce our experiments by running entry points that we created.

Directory Structure

When you unarchive the file, you will have following directory structure.

```
./
  pom.xml
  src/
    main/
      java/
        com/
          github/
            dakusui/
    test/
      java/
        com/
          github/
            dakusui/
              peerj/   ①
                CasaExperimentSimple.java
                CasaExperimentSuite.java
                SyntheticModelSuiteForIncrementalGenerationWithActs.java
                SyntheticModelSuiteForIncrementalGenerationWithPict.java
                SyntheticModelSuiteForIncrementalGenerationWithWPJoin.java
                SyntheticModelSuiteForScratchGenerationWithActs.java
                SyntheticModelSuiteForScratchGenerationWithJCUnit.java
                SyntheticModelSuiteForScratchGenerationWithPict.java
                SyntheticModelSuiteForScratchGenerationWithWPJoin.java
                SyntheticModelSuiteForScratchGenerationWithWPJoinBasedOnJCUnit.java
                SyntheticModelSuiteForScratchGenerationWithWPJoinBasedOnPict.java
      resources/
        bin/
          {Your OS Name}  ②
            pict
          acts_3.0.jar    ③
        testresults/
          CHOICE_ORDER_IMPROVEMENT/ ④
          Summary/ ⑤
  target/ ⑥
    acts/
    pict/
```

[1]: A directory that stores entry points to conduct experiments. [2]: A directory to store a PICT executable. We bundled PICT that we compiled on our local environment for macOS. [3]: Place your ACTS executable jar file here. [4]: A directory that stores our raw experiment results. [5]: Spreadsheets that summarized our experiment results are stored here. [6]: Your experiment results are stored under the directory of the engine name (`acts` or `pict`) you used.

Maven-based Java project

Installation and Execution

1. Unarchive the `.zip` file somewhere in your file system.
2. Place ACTS executable jar file in an appropriate directory.
3. Place PICT executable binary in an appropriate directory if necessary.
4. Import the `pom.xml` to your IDE.
5. Run one of the entry pont classes as a JUnit test.

ACTS

ACTS is not an open source software product, and you need to acquire its binary from here by its site owner.

```
https://csrc.nist.gov/Projects/automated-combinatorial-testing-for-software
```

Place the executable jar file under `src/test/resources/bin` directory as `acts_3.0.jar`.

PICT

We have conducted our experiments on macOS and bundled PICT binary we used in the archive file. When you are going to reproduce the experiments on other OSes, you need to prepare the binary by yourself.

You can clone the code and you can build an executable binary of PICT for your OS by following the instruction found there.

```
https://github.com/microsoft/pict
```

The directory to place the binary is as follows.

```
src/test/resource/bin/{Your OS Name}
```

`{Your OS Name}` can be figured out by your JVM's system property `os.name`.

Entry points

Directly under the directory `src/test/java/com.github.dakusui.peerj`, entry points that are used in our experiments are provided.

Entry points for CASA data models

There are a couple of entry points, which are `CasaExperimentSimple` and the other is `CasaExperimentSuite`. The former is useful for checking if the installation is working fine. The latter contains the following inner classes, each of which can be used as an independent entry point.

- Strength2
- Strength3
- etc.

Entry points for Synthetic models

Entry points for our synthetic model experiments are named SyntheticModelSuite{Incremental,Scratch}With{Acts,Pict,WPJoin}. Inside them, inner classes are defined to execute

Each of these inner classes can be used as an independent entry point. For instance, SyntheticModelSuiteForScratchGenerationWithActs has following inner classes, that you can use as independent entry points.

- Debug
- Strength2
- Strength3
- Strength4
- etc.

Details about the CASA data models

They are found under the directory src/test/resources/models/Real/2way.

- APACHE (172 factors, 2-4 levels, 7 predicates)
- BUGZILLA (51 factors, 2 levels, 5 predicates)
- GCC (199 factors, 2 levels, 40 predicates)
- SPINS (18 factors, 2-4 levels, 13 predicates)
- SPINV (55 factors, 2-4 levels, 49 predicates)
- TCAS (12 factors, 2-10 levels, 3 predicates)

You can find the definition of the format here. Note that the files contain the information about the strength (t), the tools we provide ignore it and use the strength explicitly for our experiments.

Experiment Results

Under the directory src/test/resources/testresults, our experiment results are stored.

Summary Spread-sheets

Under the directory src/test/resources/testresults/Summary, there are two spread-sheets,

- peerj-wpjoin-result.xlsx: A spread-sheet that summarizes the raw experiment results.
- peerj-wpjoin-result-summary.xlsx: A spread-sheet that we used for anlayzing the experiment

results.

Raw experiment results

Under the directory `src/test/resources/testresults/CHOICE_ORDER_IMPROVEMENT`, raw experiment results are found. Each directory has a name which describes stringth, use case scenario, data model, and optionally generation engine.
