# Supplementary material for: Accelerating covering array generation by combinatorial join for industry scale software testing
Source: PeerJ Comput Sci. 2022 Feb 11;8:e720. doi: 10.7717/peerj-cs.720 (PMC9044240; doi:10.7717/peerj-cs.720)
Supplement: Supplemental Information 2 — The data set used in the experiments and the results of the experiments. [file peerj-cs-08-720-s002.zip › experiment/src/site/docs/ThePipeline.pptx]

## Slide 1
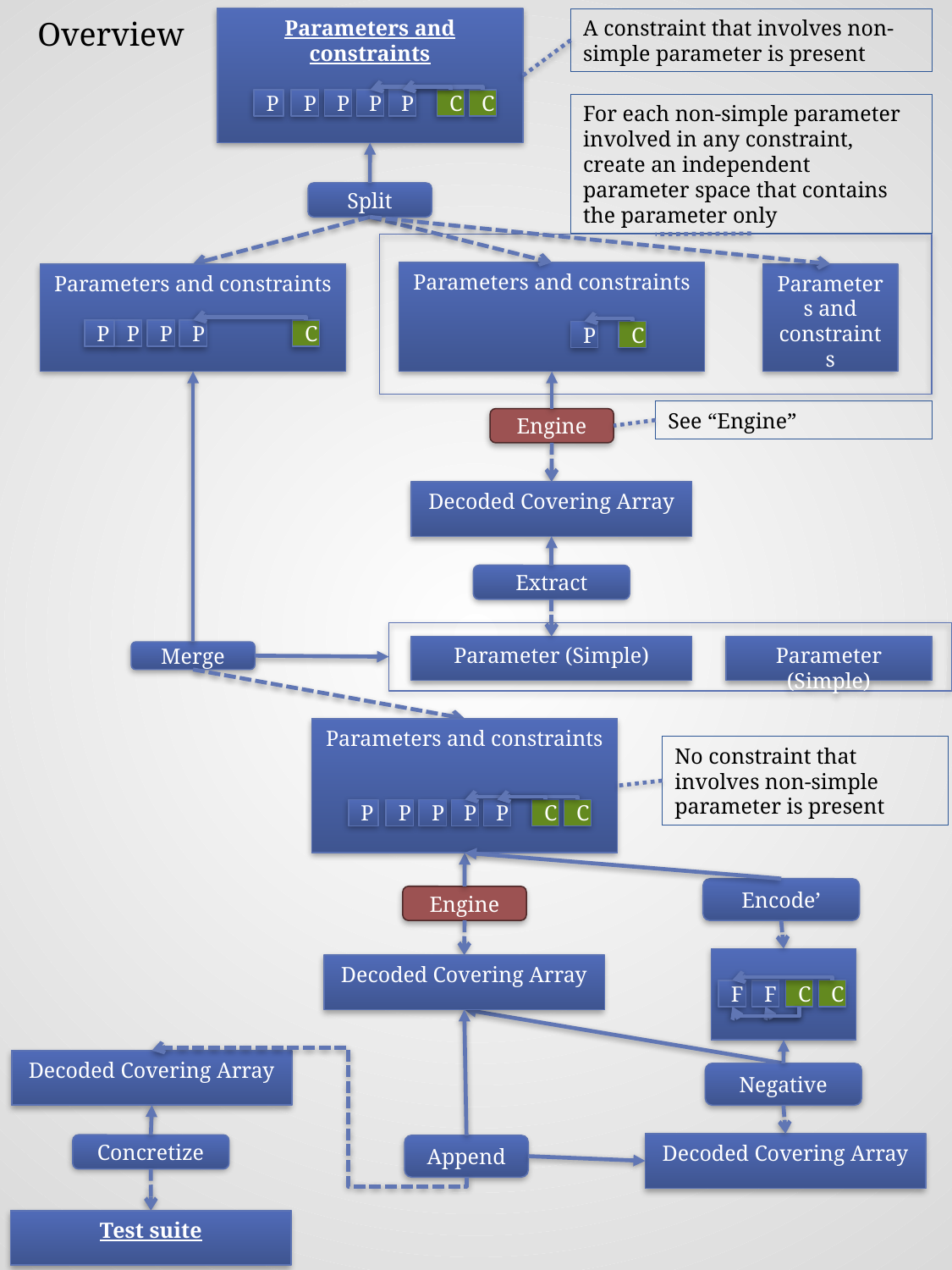

Overview
Parameters and constraints
C
C
P
P
P
P
P
A constraint that involves non-simple parameter is present
For each non-simple parameter involved in any constraint, create an independent parameter space that contains the parameter only
Split
Parameters and constraints
C
P
Parameters and constraints
Parameters and constraints
C
P
P
P
P
See “Engine”
Engine
Decoded Covering Array
Extract
Parameter (Simple)
Parameter (Simple)
Merge
Parameters and constraints
C
C
P
P
P
P
P
No constraint that involves non-simple parameter is present
Encode’
Engine
C
F
C
F
Decoded Covering Array
Decoded Covering Array
Negative
Decoded Covering Array
Concretize
Append
Test suite

## Slide 2
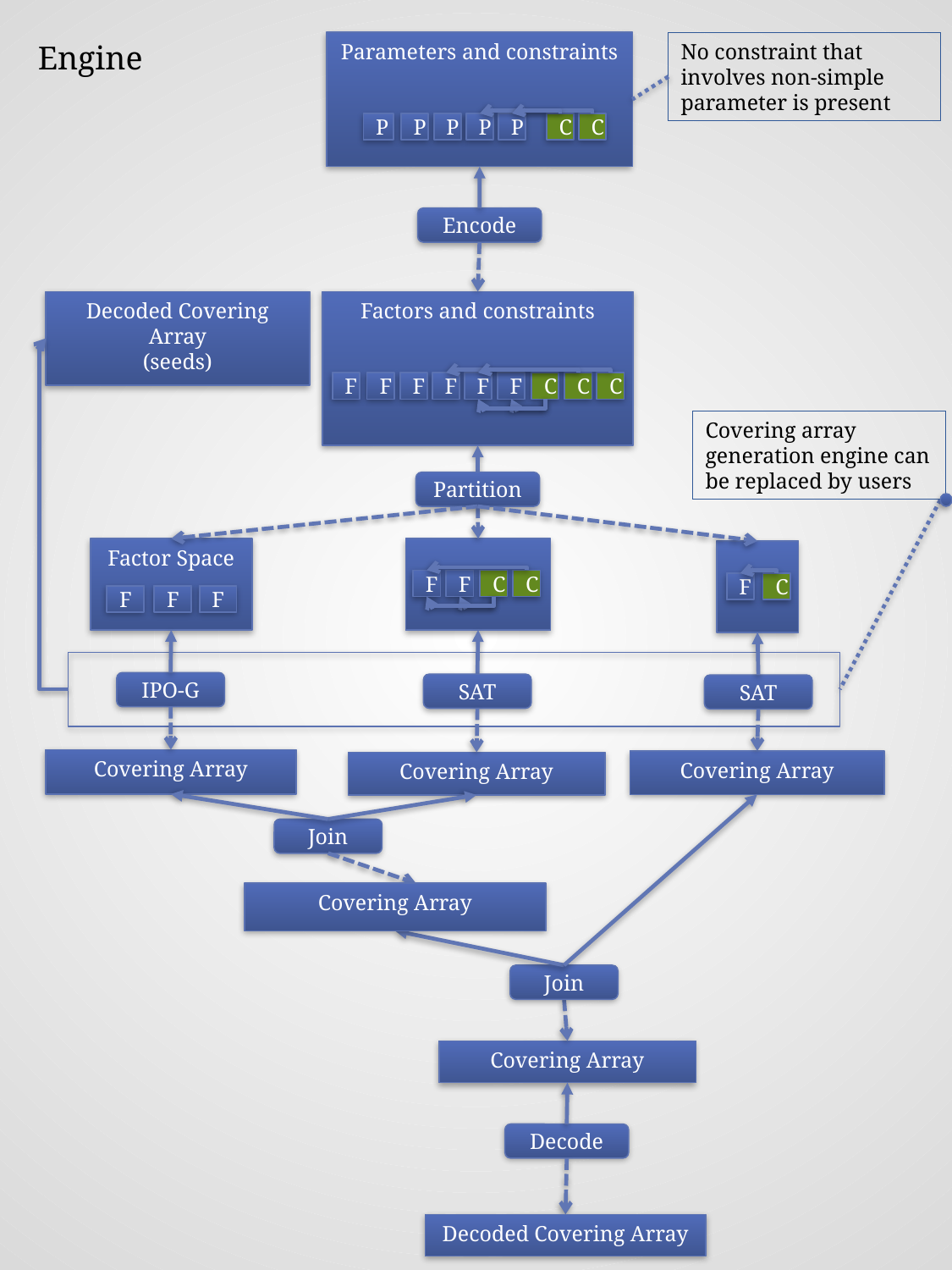

Engine
Parameters and constraints
C
C
P
P
P
P
P
No constraint that involves non-simple parameter is present
Encode
Decoded Covering Array
(seeds)
Factors and constraints
C
C
F
C
F
F
F
F
F
Covering array generation engine can be replaced by users
Partition
C
F
C
F
Factor Space
F
F
F
C
F
IPO-G
SAT
SAT
Covering Array
Covering Array
Covering Array
Join
Covering Array
Join
Covering Array
Decode
Decoded Covering Array
