# Supplementary figures and images for: Accelerating covering array generation by combinatorial join for industry scale software testing
Source: PeerJ Comput Sci. 2022 Feb 11;8:e720. doi: 10.7717/peerj-cs.720 (PMC9044240; doi:10.7717/peerj-cs.720)

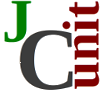

Supplement: Supplemental Information 2 — The data set used in the experiments and the results of the experiments. [file peerj-cs-08-720-s002.zip › experiment/src/main/resources/JCunit-logo-compact.png]

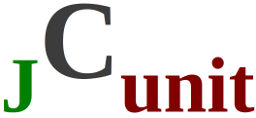

Supplement: Supplemental Information 2 — The data set used in the experiments and the results of the experiments. [file peerj-cs-08-720-s002.zip › experiment/src/main/resources/JCunit-logo.png]

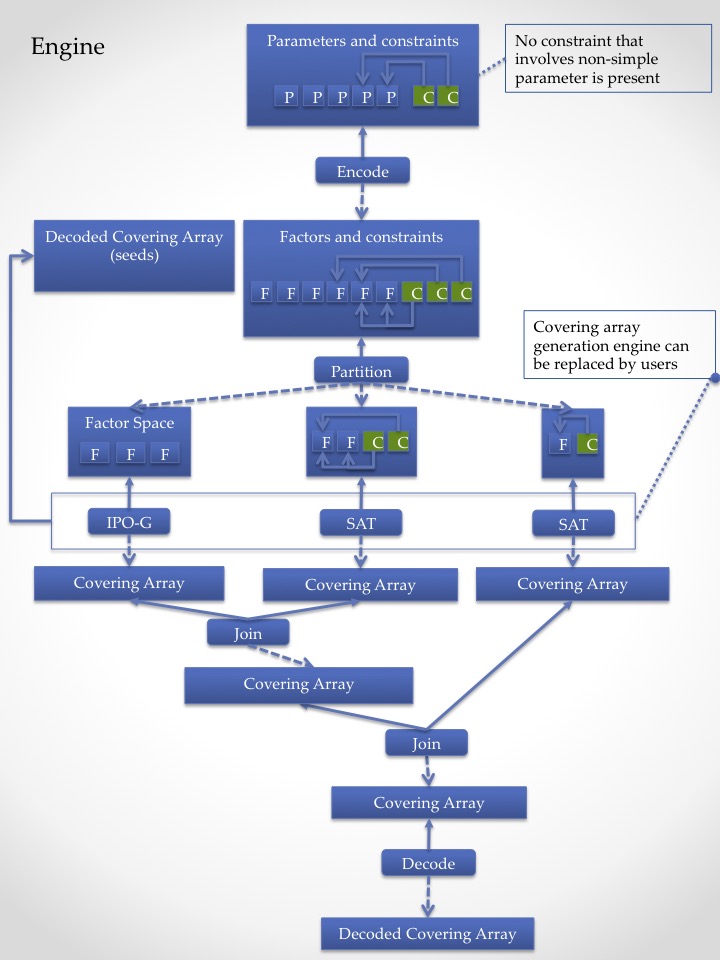

Supplement: Supplemental Information 2 — The data set used in the experiments and the results of the experiments. [file peerj-cs-08-720-s002.zip › experiment/src/site/docs/ThePipeline/Slide2.jpg]

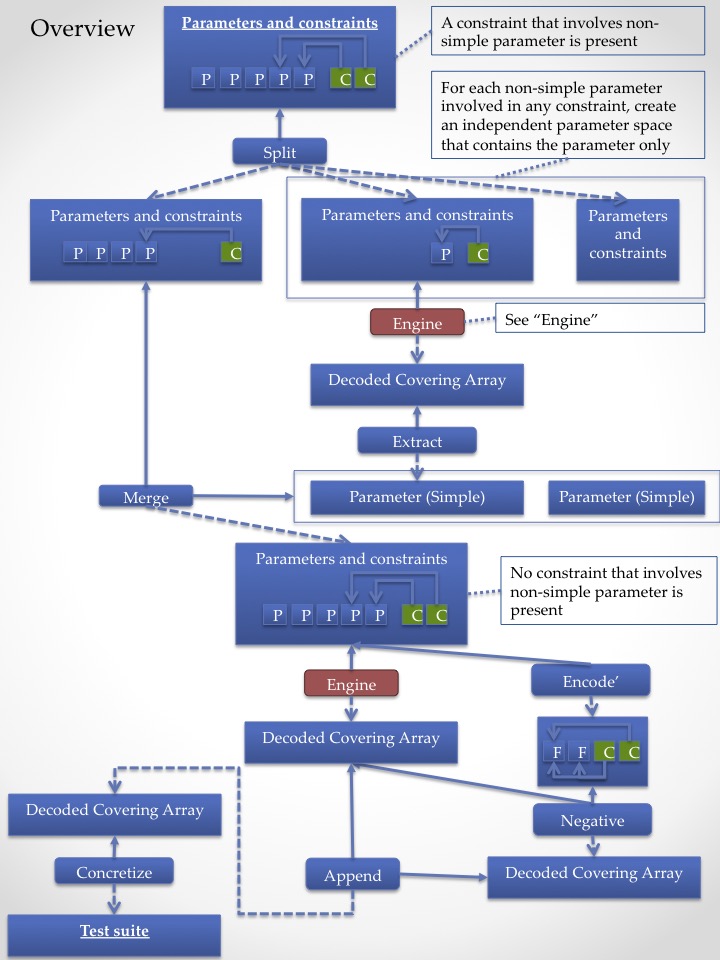

Supplement: Supplemental Information 2 — The data set used in the experiments and the results of the experiments. [file peerj-cs-08-720-s002.zip › experiment/src/site/docs/ThePipeline/Slide1.jpg]

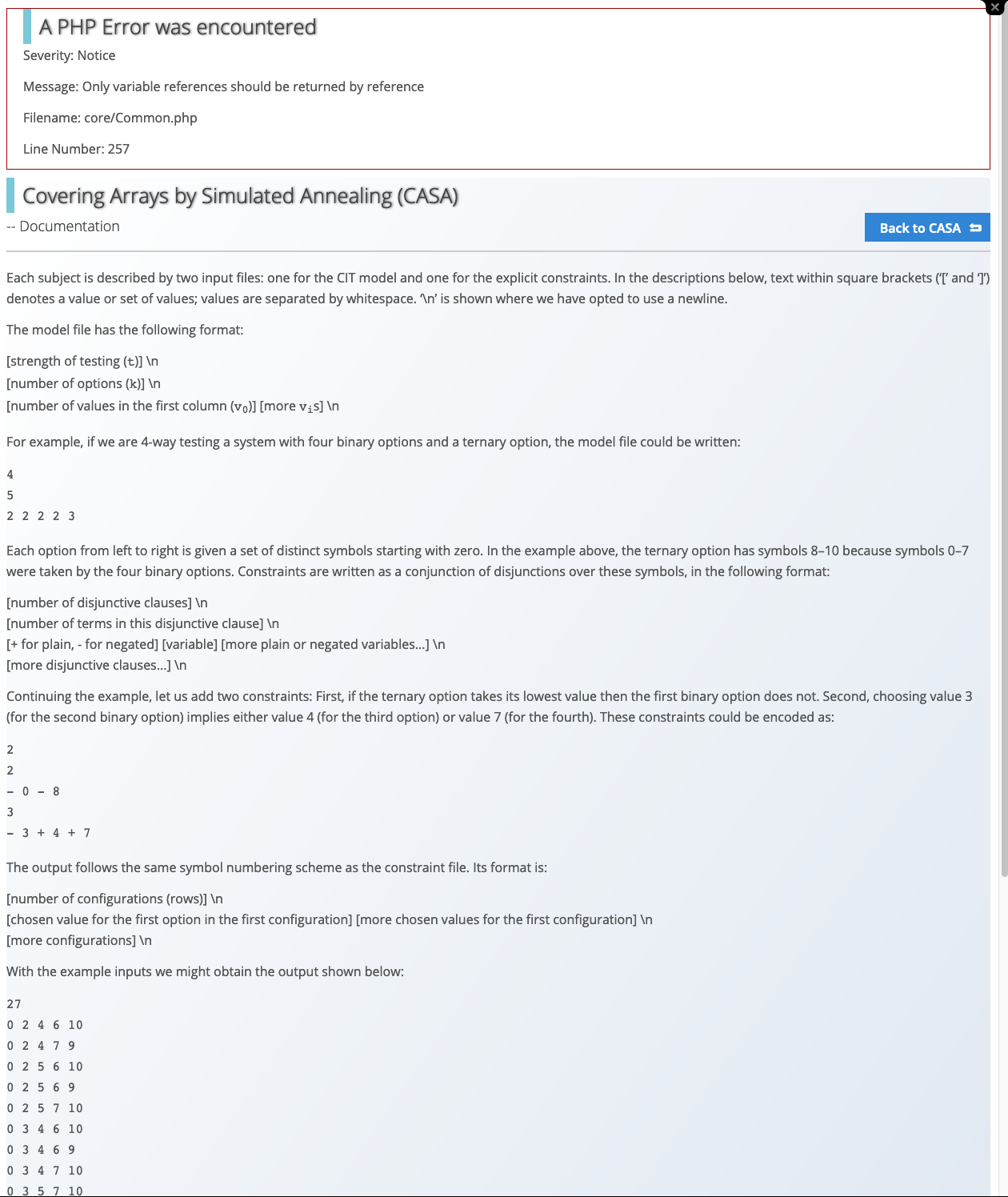

Supplement: Supplemental Information 2 — The data set used in the experiments and the results of the experiments. [file peerj-cs-08-720-s002.zip › experiment/src/test/resources/models/format/FORMAT.png]

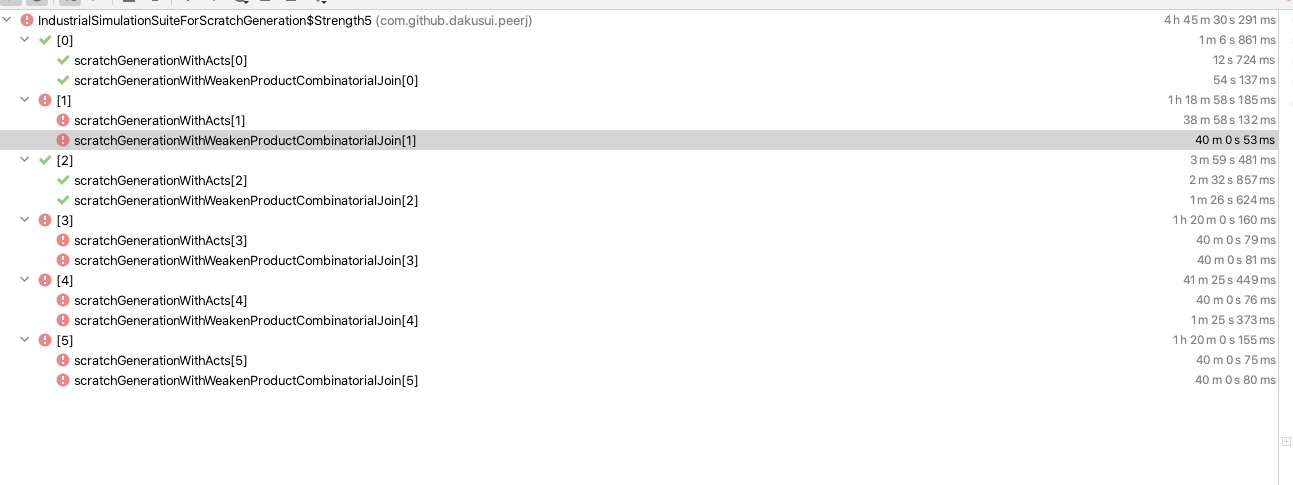

Supplement: Supplemental Information 2 — The data set used in the experiments and the results of the experiments. [file peerj-cs-08-720-s002.zip › experiment/src/test/resources/testresults/CHOICE_ORDER_IMPROVEMENT/images/Screen Shot 2021-02-06 at 2.45.32.png]
